# Molecular Imprinting Technology for Advanced Delivery of Essential Oils

**DOI:** 10.3390/polym16172441

**Published:** 2024-08-28

**Authors:** Greta Kaspute, Arunas Ramanavicius, Urte Prentice

**Affiliations:** 1 Department of Nanotechnology, State Research Institute Center for Physical Sciences and Technology (FTMC), Sauletekio Av. 3, LT-10257 Vilnius, Lithuania; 2 Department of Personalised Medicine, State Research Institute Centre for Innovative Medicine, Santariskes St. 5, LT-08410 Vilnius, Lithuania; 3 Department of Physical Chemistry, Institute of Chemistry, Faculty of Chemistry and Geosciences, Vilnius University, Naugarduko St. 24, LT-03225 Vilnius, Lithuania; arunas.ramanavicius@chf.vu.lt

**Keywords:** drug delivery systems, essential oils, molecular imprinting, challenges, therapeutic perspectives

## Abstract

Essential oils (EOs) hold therapeutic potential, but their conventional delivery systems have some limitations. This review focuses on the critical review and discussion of research related to EO delivery systems. The review also explores how molecular imprinting technologies (MIT) can advance EO delivery. MIT offer several techniques, namely covalent, non-covalent, and semi-covalent imprinting, creating targeted cavities that selectively bind and release EOs. These approaches promise significant advantages including increased selectivity, controlled release, and protection from environmental degradation. However, some challenges related to the stability and biocompatibility of MIPs remain unsolved. Integrating nanotechnology through methods like nanoparticle imprinting and some lithographic techniques seems promising to overcome these limitations. Some recently established models and systems used for EO-related research are paving the way for a more efficient and targeted EO delivery approach to harnessing the therapeutic power of EOs. Therefore, some recent and future research seems promising, and eventually it will increase the effectiveness of MIP-based EO delivery systems.

## 1. Introduction

Recently, essential oils (EOs) have become popular among people for various reasons, with the main one being dermal exposure [1,2]. EOs are alternatively referred to as essences, volatile oils, etheric oils, or aetheroleum, consisting of natural blends of volatile, lipophilic, and fragrant compounds typically present in aromatic plants [3]. These substances consist of active compounds which impact EOS’ biological activities, i.e., anti-microbial, anti-oxidant, anti-inflammatory, and anti-cancer effects [4]. For this reason, EOs get more attention as active substances in drug development [5,6].

Molecular imprinting technologies (MIT) involve the development of artificial recognition sites within polymeric matrices that mimic the size, shape, and spatial arrangement of functional groups found in the template molecule [7]. MIT can be implemented in different methods such as bulk imprinting, surface imprinting, and epitope imprinting techniques [7]. Molecularly imprinted polymers (MIP) can be easily synthesized as effective substitutes for antibodies and enzymes across multiple assays [8], having various formats for different medical purposes, such as targeting, imaging, diagnostics, and biomarker detection [9,10,11,12,13]. These synthetic polymers are designed to mimic the function of antibodies by interacting with specific antigens. During polymerization, MIPs selectively bind to molecules that serve as templates [14]. They offer benefits similar to the antibody–antigen interactions, including high specificity and selectivity [15], and are cost-effective to produce [14]. The study of biological samples (urine, blood, saliva) faces challenges like complex matrices, diverse components, and low concentrations of active ingredients [16]. Therefore, in healthcare systems, the use of commercially available sensors with validated properties is essential [16]. Biologically applied MIPs can target antigens, which can be valuable in personalized medicine and can be used as tools for analytics, diagnostics, and drug delivery systems (DDS) [9]. 

The objective of this review is to comprehensively examine the potential applications of MIT for incorporating EOs as components of drug delivery systems. To develop safe and efficient EO delivery systems, it is imperative to explore the utilization of nanotechnology tools, and determine any associated toxicity, biodistribution, and pharmacokinetics.

## 2. Essential Oils’ Active Substances as Part of MIP

The drug delivery approach for herbal medicine lacks efficiency and causes adverse effects from diverse compounds. Novel delivery systems could save phyto-constituents from internal and external factors. Nanotechnology-driven formulations for herbal drugs have demonstrated encouraging outcomes such as biocompatibility and biodegradable delivery platforms (lipids, polymers, and nanoemulsions), which enhance the solubility, stability, bioavailability, and pharmacological efficacy of herbal medicines [17].

EOs are insoluble in water, but soluble in alcohol, ether, and fixed oils. Additionally, recent findings have suggested that EOs primarily penetrate the outermost layers of the skin, potentially enhancing epidermal water balance through a “filmogenic” mechanism. Moreover, due to lipid solubility, EO compounds can transfer through the blood–brain barrier, accessing the fluids surrounding the brain [17]. Most EOs are colourless or light yellow liquids at room temperature, and chemical components generally have a molecular weight below 300 [3].

Recently, EOs have not been directly used in MIP development. However, some key chemical properties are relevant to create these synthetic recognition materials. The selection of template and monomer molecules is crucial for defining the specificity and properties of MIPs, with combinatorial and computational screening methods aiding this process. Typically, MIPs are synthesized using a single functional monomer [18]. Ramstroem et al. demonstrated that MIPs with dual functional monomers outperform those synthesized with a single monomer, owing to the synergistic and complementary effects of using two monomers [19]. Functional monomers are selected to interact specifically with the template molecule, forming a stable template–monomer complex essential for effective molecular recognition. After copolymerization with cross-linking monomers, a macroporous matrix with microcavities complementary to the template is developed. Once the template is removed, the polymer’s binding sites selectively recognize and bind the template molecules, with the nature of these interactions defining the characteristics of the binding sites and the classification of molecular imprinting [20]. Functional monomer development is associated with the building blocks of the MIP, which are small organic molecules chosen based on their ability to interact with the target molecule through specific functional groups [21]. Examples include amines (NH_2_), carboxylic acids (COOH), alcohols (OH), amides (CONH_2_), and aromatic rings (containing benzene groups) [21]. These groups form specific interactions (like hydrogen bonding, ionic interactions, or pi-pi stacking) [22] with the target molecule. The selection of functional monomers is crucial for determining the selectivity and binding affinity of the final MIP towards the target molecule [21]. Hydrogen bonds, hydrophobic interactions, and electrostatic interactions are the most commonly utilized bonds in MIP manufacturing due to their exceptional adaptability [22]. For example, geraniol, which can be found in various EOs such as rose, palmarosa, and ninde oil [23], can interact with the active sites of cyclooxygenase and 5-lipoxygenase via hydrogen bonding and hydrophobic interactions, with its single hydroxyl group being crucial for inhibiting the target enzymes [24]. Lu et al. created novel dual-template MIPs using a straightforward precipitation polymerization method with norfloxacin and enrofloxacin as templates, enabling simultaneous selective recognition and extraction of these fluoroquinolones. The resulting polymers demonstrated high adsorption capacity and selectivity for both compounds, with effective extraction and accurate analysis of the fluoroquinolones from various water samples, demonstrating the potential for enhanced applications in dual-template imprinting research [25].

Cross-linkers are bifunctional molecules that link the functional monomers together, forming a stable three-dimensional network [21]. The reaction mechanism of molecules is determined by the length and structure of the linker connecting the two monomers, which has an impact on the activity of the molecules [26]. Common cross-linkers include ethylene glycol dimethacrylate (EGDMA), 1,4-diaminobutane (DAB), and divinylbenzene (DVB) [27]. Cross-linkers define the pore size and rigidity of the MIP network. The pore size needs to be large enough for the target molecule to access the binding sites within the MIP [21]. Ahmadi et al. [28] introduced MIP for 1,8-cineole, which was synthesized using hydroxyl-functionalized multiwall carbon nanotubes for the selective extraction of 1,8-cineole from water distillates of *Artemisia sieberi* (sagebrush) and thyme. The MIP synthesis involved using 1,8-cineole as the template, methacrylic acid as the functional monomer, ethylene glycol dimethacrylate as the cross-linker, and benzoyl peroxide as the initiator, with key parameters optimized to enhance synthesis and extraction efficiency [28]. The MIP demonstrated a detection limit of 0.04 μg/mL, a dynamic linear range of 0.125–100 μg/mL, relative standard deviations of 1.45–4.3% for samples spiked at 1 and 70 μg/mL, and relative recoveries between 93.8 and 98.2% [28].

Solvents dissolve the reaction mixture containing the functional monomers, cross-linkers, and initiators during MIP synthesis [27]. Common solvents include porogens like toluene, chloroform, and acetonitrile [27]. Several studies have identified that EOs can contain small amounts of toluene, e.g., the EO of *Ferulago angulata* [29], *Sinapis arvensis* [30], and *Genipa americana* [31] were found to contain 0.1% toluene. Porogens are often identical to the solvent and play a crucial role in creating pores within the final MIP material [27]. The porogen molecules are removed after polymerization, leaving behind a network with cavities that can accommodate the target molecule. The choice of solvent and porogen affects the overall porosity, pore size distribution, and accessibility of the binding sites within the MIP [27].

Additional properties consist of the monomer/cross-linker ratio which influences the density of cross-linking and consequently the rigidity and selectivity of the MIP [27,32]. An initiator is what triggers the polymerization reaction between the functional monomers and cross-linkers [27]. A recent study by Sarmast et al. [33] introduced a cross-linked film containing a blend of EOs and silver nanoparticles (AgNPs) that was created for use as active packaging to extend meat’s shelf life. Results showed that incorporating 0.75% R (*w*/*w*) and 5 kGy irradiation improved the film’s tensile strength, water insolubility, and water barrier properties, though it decreased film elongation, indicating a more compact and cross-linked structure [33]. Infrared spectroscopy confirmed that appropriate irradiation dosages produced cross-linking bonds in the G film, forming a denser network, and microbial analyses demonstrated that the EO-AgNP-incorporated film exhibited anti-microbial activity against spoilage and pathogenic bacteria, effectively extending meat’s shelf life by up to 21 days, making it a promising biodegradable packaging option for the food industry [33]. Other research microencapsulated orange EO using complex coacervation with whey protein isolate (WPI) combined with carboxymethylcellulose, sodium alginate, and chitosan, optimizing conditions based on pH, protein ratio, and solid concentration [34]. The highest encapsulation efficiency (EE) for wet microcapsules was 88–94%, with freeze-dried microcapsules maintaining over 80% EE but forming a solid cake, while spray-dried microcapsules, particularly WPI and WPI, cross-linked with tannic acid and transglutaminase, respectively, achieved the highest EE of 47% and 50%, a 400% improvement over non-cross-linked samples [34].

Recently, MIPs have been more likely to be used as extraction methods for EOs. For example, MIP solid-phase extraction employs MIPs as sorbents to address the limited specificity of conventional solid-phase extraction methods. This leads to straightforward preparation, robust stability, and reusability. This technique can selectively isolate quercetin or its structural analogues from complex matrices while concentrating the target analyte efficiently [35]. Another study by Kasiri et al. introduced a magnetic MIP-dispersive solid-phase microextraction (MMIP-DSPME) method combined with high-performance liquid chromatography-ultraviolet (HPLC-UV) for the determination of thymol and carvacrol in pharmaceutical syrups [36]. Optimization using the design of experiments and response surface methodology identified key parameters such as MMIP mass, sample pH, eluent type and volume, and sorption and elution times as being critical for maximizing analyte extraction recovery [36]. Under optimal conditions, the method achieved a limit of detection of 0.042 ng mL^−1^, a limit of quantification of 0.140 ng mL^−1^, and demonstrated high sorption capacities for thymol (64.1 mg g^−1^) and carvacrol (72.6 mg g^−1^) [36].

Various MIPs are adaptable as DDS models due to benefits associated with selective recognition, increased drug loading capacity, sustained release capability, and durability under challenging conditions [22,37]. Additionally, MIPs’ ability to sustain release and their flexibility in surface modification, enabling targeted delivery through various stimuli-responsive mechanisms, makes them desirable in DDS [38]. These include external stimuli such as magnetic fields and light, as well as internal stimuli like pH, temperature, redox conditions, and biological signals. The combined properties of sustained release and targeted delivery offered by MIPs can significantly enhance the therapeutic efficacy of drugs, particularly in applications targeting tumours [39]. EOs can play a crucial role in DDS when used as carrier matrices [40,41,42,43]. For example, Krishnaiah et al. [42] investigated the effect of different solvent systems and concentrations of menthol on the permeation of ondansetron hydrochloride through the rat epidermis. Solubility tests identified a 60% *v*/*v* ethanol–water system as having the highest permeation rate [42]. This led to the development of hydroxypropyl cellulose gel formulations containing various concentrations of menthol [42]. In vitro and in vivo research has identified enhanced transdermal delivery of zidovudine using novel chemical enhancers such as t-anethole, carvacrol, thymol, and linalool, with L-menthol serving as a reference enhancer [40]. The use of an isopropyl alcohol/water solvent has generally resulted in superior absorption compared to propylene glycol/water when combined with most enhancers [40]. These findings underscore the potential of these enhancers to facilitate effective transdermal delivery of the drug [40]. Esmaeili et al. [43] developed an oil-in-water (O/W) nanoemulsion formulation of eugenol which demonstrated markedly improved anti-inflammatory activity compared to a commercially available piroxicam gel in a rat model of carrageenan-induced paw edema [43]. Higher concentrations of eugenol did not correlate with increased anti-inflammatory effects. Furthermore, nanoemulsions containing piroxicam exhibited reduced anti-inflammatory efficacy compared to formulations lacking piroxicam [43].

## 3. Opportunities to Apply Molecular Imprinting Technologies for the Delivery of Essential Oils

Molecular imprinting is a technique of specific functional monomers assembling around a template molecule, followed by polymerization in the presence of a crosslinker [8]. Sensitive transduction of analytical signals is possible with physical methods, but achieving reliable selectivity in bioanalytical systems remains challenging. New chemical materials and technologies have been developed to address this. Although various semiconducting materials are used for sensor signal transducers, conducting polymers (CPs) are the most frequently applied [44], e.g., a molecularly imprinted polypyrrole layer to target-DNA [45,46], detect *Listeria monocytogenes* [47], methylene blue [48], SARS-CoV-2 spike glycoprotein [12], bovine leukemia virus glycoproteins [49], glyphosate [50], and bisphenol [51], as well as theophylline determination [52]. Additionally, other popular types of CPs are polythiophene(PTH), poly(3,4-ethylenedioxythiophene) [53], and polyaniline (PANI), which can be used as a formate layer to sensitize l-tryptophan [13].

### 3.1. The Most Promising Types of Molecular Imprinting Technology

**Figure 1:**
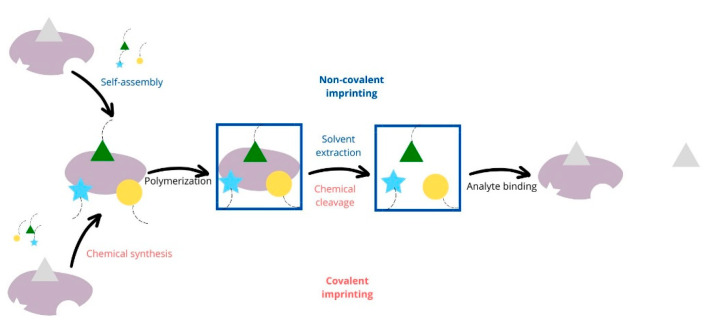
Non-covalent and covalent imprinting schematic representation. In both cases, the various functional monomers (different shapes—stars, triangles, and circles represent different structures) are selected to interact with the functional groups of the imprint template (grey cloud) and are polymerized while the template is present. This process develops specific binding sites through covalent or non-covalent interactions between the monomer and the template (frame), followed by crosslinked co-polymerization.

The wide use of MIPs is the reason that their synthesis methods are essential, encompassing three strategies: covalent, non-covalent, and semi-covalent approaches [54] (Figure 1).

The non-covalent approach is most prevalent due to its simplicity, where the target is removed from the MIP [54]. In this method, the template and functional monomer form an in situ complex through non-covalent interactions like hydrogen bonding, electrostatic forces, van der Waals forces, or hydrophobic interactions [55]. Synthesizing MIPs is straightforward and cost-effective, involving the mixing of functional monomers, templates, cross-linkers, and initiators in an appropriate solvent [55]. This approach offers numerous advantages, including easy preparation, the removal of the template–monomer complex, the rapid binding of templates to MIPs, and the broad applicability to various target molecules [55]. However, to ensure the optimal formation of the labile template–monomer complex and to minimize non-specific binding sites, careful selection of polymerization conditions is crucial [55]. For example, non-covalent molecular imprinting of sterols has been challenging, primarily because sterols typically have only one hydrogen bond acceptor site and are low in polarity, limiting their capacity to form strong association complexes with complementary functional monomers [56]. Low-temperature polymerization reactions, usually at 4 °C, have been necessary for the non-covalent preparation of cholesterol-imprinted polymers [56].

The covalent strategy involves reversible covalent bonds between the monomer and template, with target rebinding relying on the formation or breaking of these bonds [54]. The covalent approach is based on reversible covalent bonds, introduced by Wulff in 1995 [38,57]. This method includes forming a covalent bond between the template and monomer, which is cleaved during polymerization to remove the template from the MIP matrix. Rebinding the template recreates the same covalent linkage, but the need for rapidly reversible covalent interactions limits suitable templates, and the robust nature of these interactions can hinder the achievement of thermodynamic equilibrium due to slow dissociation and binding [38]. Şenocak et al. [58] developed a catechin sensor using single-walled carbon nanotubes (SWCNTs) covalently functionalized with terminal ethynyl-bearing subphthalocyanine (SubPc) to create a new hybrid material, SWCNT-SubPc, via a “click” reaction [58,59]. This sensor demonstrated a 2,2 and eight-fold increase in differential pulse voltammetry responses to catechin compared to SWCNT-modified glassy carbon electrodes (GCE) and bare GCE, respectively, in Britton–Robinson buffer solution (pH 3) [59]. When tested on real samples of green, black, and fruit teas, the sensor showed a lower limit of detection (13 nM) and a broader dynamic range (0.1–1.5 μM) than most previously studied electrodes, along with high stability and repeatability in catechin determination [59].

The semi-covalent method combines the advantages of both approaches, using covalent interactions during synthesis and non-covalent interactions for target rebinding. Two primary mechanisms for MIP synthesis are free-radical polymerization and the sol-gel process, with the former including various techniques such as suspension, bulk, and microwave polymerization, and the latter involving methods like embedding and multi-stage imprinting [54]. The template of this method is initially bound covalently and then, after removal, rebinding occurs through non-covalent interactions [38]. This method combines the high affinity of covalent binding with the mild operational conditions of non-covalent rebinding, providing a balanced alternative [38,60]. This method using a carbonyl group as a sacrificial spacer was utilized to synthesize MIPs for phenols. The optimal MIP was prepared using 4-chlorophenyl (4-vinyl)phenyl carbonate as the template, EGDMA as the cross-linker, 2,2-azobisisobutyronitrile (AIBN) as the initiator, and chloroform as the porogen. This semi-covalently imprinted polymer demonstrated superior selectivity for phenols compared to its non-covalently imprinted counterpart, and showed reduced peak broadening and tailing, indicating its potential as a stationary phase for the quantitative determination of phenols [61].

### 3.2. Kinetics’ Role in EO Integration in MIP

Drug release kinetics is crucial for evaluating the clinical viability of drugs, the therapeutic effectiveness of nanocarriers, and their potential use in clinical settings. Typically, to assess drug release kinetics, a drug release profile is generated by plotting the ratio of the released drug to the total drug encapsulated in the nanocarrier over time, using methods such as sample-and-separate, membrane-barrier, continuous-flow, and various in situ techniques [62]. The tailing of the template peak on MIPs has been linked to the wide distribution of adsorption site energies and slow intraparticle mass transfer kinetics [63]. The adsorption model is a crucial tool for investigating the adsorption mechanism. Common models include adsorption isotherms, adsorption kinetics, and adsorption thermodynamics [64]. To enhance MIPs’ chromatographic performance, researchers have explored creating a more homogeneous surface through chemical modifications and imprinting within dendrimers, as well as improving binding site accessibility by developing imprinted polymers that position these sites exclusively on the polymer surface [63]. Liquid crystal monomers have been proposed for MIP synthesis to significantly reduce the need for chemical cross-linking. These monomers form physical cross-links through noncovalent reversible interactions, enhancing mass transfer kinetics, and have shown clear improvements in resolution and column efficiency when used as selective stationary phases in liquid chromatography and capillary electrophoresis [65].

MIPs provide customizable synthetic recognition sites that can be tailored for use in analytical, diagnostic, and drug delivery systems, advancing the development of personalized medicine [66]. In clinical practice, monitoring the drug’s durability and release in the blood is important, as initial doses are based on a patient’s size and weight, but future dosing for effective treatment can be determined by measuring blood–drug concentration and release kinetics. To achieve this, MIP-based devices can be used to track drug release profiles in human serum [62]. To enhance the accessibility of target analytes to imprinted sites, hollow MIP beads (H-MIPs) have been developed. These hollow particles allow analytes to access both the inner and outer surfaces, thereby increasing particle capacity and improving mass transfer kinetics compared to conventional MIPs or even core-shell MIPs created through surface imprinting [65]. Surface MIPs offer several advantages over traditional MIPs, including a larger specific surface area, more accessible imprinted sites, stronger binding forces, faster mass transfer, and improved binding kinetics [64].

Detecting plant volatile organic compounds (VOCs) is useful for determining harvest time, and monitoring pests and diseases in agriculture. MIPs are engineered to capture these volatiles and can serve as sensing elements. The concentration of VOCs released at various stages of fruit maturity can be used to assess the ripeness of fruits and vegetables [67]. A MIP for vanillin was successfully synthesized using multi-step swelling suspension polymerization with polystyrene (PS) as the seed. Characterization of the PS-MIP exhibited superior molecular recognition selectivity, enhanced adsorption capacity, and greater binding capacity compared to the MIP produced by bulk polymerization [68]. A quartz crystal microbalance (QCM)-MIP sensor modified with chitosan/α-pinene, after a heating process, demonstrated high selectivity and sensitivity for α-pinene. It was noted that the MIP process’s imprinting effect was significantly enhanced by using α-pinene and chitosan polymer as the template [67]. Cao et al. developed an innovative MIP for targeting resveratrol in *Polygonum cuspidatum* for the first time, using silane-coated, porous cellulose microspheres. The adsorption followed a pseudo-second-order kinetics model, and the thermodynamic equilibrium process aligned with the Langmuir model [69].

The formation of MIPs remains unpredictable due to their insoluble and complex nature, with most methods requiring polymer solutions such as solution nuclear magnetic resonance (NMR), gel permeation chromatography, and ultraviolet spectroscopy [70]. Characterization techniques for MIPs include morphology analysis using scanning electron microscopy, transmission electron microscopy, atomic force microscopy, and scanning tunnelling microscopy, as well as physical property assessment through nitrogen sorption, elemental analysis, Fourier transform infrared spectroscopy, solid-state NMR, and swelling measurements [70]. Molecular simulation and computational chemistry can predict imprinting sites but require experimental validation due to system complexity and model approximations [70]. However, these methods provide limited information about the internal structure of MIPs [70]. Additionally, as mentioned in the previous paragraph, EOs have limited physical and chemical properties. Conventional gas chromatography (GC)-flame ionization detector (FID)/mass spectrometer (MS) is a standard method for analyzing the chemical composition and quality of EOs [71]. Enhanced analytical techniques, such as multidimensional GC and new stationary phases like ionic liquids, offer improved separation capabilities and resolve complex co-elutions, reducing the need for multiple columns. Fast-GC methods and miniaturized instruments are emerging to increase sample throughput and reduce costs, though conventional methods remain prevalent in routine analysis [71]. Water-soluble molecules within the MIP system provide fast extraction with the use of magnetic materials incorporated into MIP [72].

### 3.3. Molecularly Imprinted Polymers Applied to the Development of Biosensors for the Detection of EOs

Despite the progress of DDS development over the years, there are still challenges such as severe side effects and limited bioavailability [73]. These systems involve the controlled administration of therapeutic substances to achieve therapeutic effects while minimizing side effects and toxicity [9]. Therefore, MIPs have received attention due to promising drug delivery opportunities, i.e., selective recognition capabilities, improved drug loading capacities, sustained release profiles, and resilience under adverse conditions [37]. Additionally, when loading a drug into the MIP, it binds specifically to the imprints, enabling controlled release by altering environmental factors such as pH, temperature, or ionic strength [9].

Recently, EOs became a target for clinical trials to analyse empirical knowledge in practice. For example, a randomized controlled trial in Turkey investigated the impact of lavender EO aromatherapy on sleep and fatigue in multiple sclerosis patients. Results showed a significant improvement in both sleep quality and fatigue levels after the intervention, with the Fatigue Severity Scale and Pittsburgh Sleep Quality Index scores notably reduced (*p* < 0.001) [74]. Wakui et al. implemented a placebo-controlled, randomized trial that explored the effects of bergamot EO on psychological stress, sleep quality, and morning wakefulness during the COVID-19 pandemic. The results showed that bergamot EO significantly improved sleep quality, mood, and morning alertness, and reduced depression, anxiety, and stress among university students [75]. A randomized clinical pilot trial by Lucena et al. investigated the effects of lavender EO and sleep hygiene guidance on sleep patterns, quality of life, and hot flashes in post-menopausal women with insomnia. Participants in the aroma group, who inhaled lavender EO before bedtime, showed significant improvement in overall quality of life and self-reported sleep metrics. Although both groups benefited from sleep hygiene guidance, no significant differences were observed in hot flush frequency or daytime sleepiness [76]. Another clinical study evaluated the impact of chamomile EO aromatherapy on anxiety and hemodynamic indices in Acute Coronary Syndrome patients. The double-blind, randomized trial involved 154 patients, with the intervention group inhaling chamomile EO and the placebo group inhaling sesame oil for two nights. Results showed that the aromatherapy significantly reduced anxiety levels, systolic and diastolic blood pressure, and heart rate in the intervention group compared to the placebo group (*p* < 0.001) [77]. A randomized controlled trial compared the effects of damask rose and chamomile EO aromatherapy on pre-operative anxiety and pain in patients awaiting emergency orthopaedic surgery. Ninety participants were randomized into three groups, with the intervention groups inhaling damask rose or chamomile oil. Both EOs significantly reduced the anxiety and pain levels of those in the intervention group compared to the levels of those in the control group (*p* < 0.001). Notably, damask rose reduced anxiety more quickly than chamomile immediately after the intervention (*p* = 0.01), although both oils had similar effects an hour later [78].

**Figure 2:**
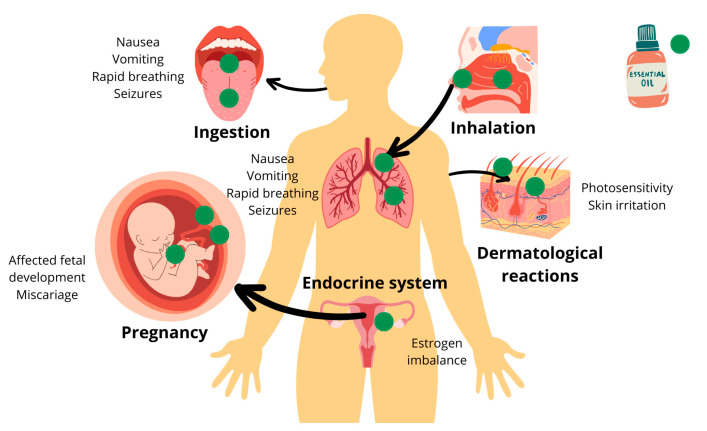
Toxicology and safety issues of EOs. Green dots represent EO’s molecules.

Due to their high content of phenolic compounds, particularly flavonoids, plant extracts like *Scutellaria baicalensis* and *Yucca filamentosa* exhibit anti-oxidant properties by neutralizing free radicals and reactive oxygen species, making them useful for reducing the toxicity of cytostatic drugs and treating various diseases, including cardiovascular and neurodegenerative conditions [79]. However, EOs face challenges such as hydrophobicity, instability, high volatility, and potential toxicity, which limit their use [80]. Encapsulation within delivery systems has proven effective in overcoming these limitations by enhancing bioavailability, improving chemical stability, and reducing the volatility and toxicity of EOs [6,80]. Despite the beneficial effects of EOs, it is crucial for users to be aware of potential negative and harmful effects when incorporating natural compounds into their diets or medicinal practices [81]. These effects can include neurological disruptions and teratogenic activity, caused by a diverse group of compounds found naturally in leaves, fruits, roots, and flowers of both toxic and non-toxic plants [81]. Notably, species within the *Lamiaceae* family, such as *Melissa*, *Mentha*, *Ocimum*, and *Origanum*, which are rich in monoterpenes, are particularly widespread and utilized in everyday applications [81]. Alternative in vitro methods and in vivo models including *Caenorhabditis elegans*, the hen’s egg test, were used to thoroughly investigate the acute, developmental, reproductive toxicity, and mucous membrane irritation, caused by commonly used rosemary, citrus, and eucalyptus EOs [82]. All EOs tested demonstrated comparable impacts against the measured parameters, with rosemary oil exhibiting slightly higher toxic potential [82]. Gene expression analysis indicated upregulation of xenobiotic and oxidative stress-related genes in response to EO exposure. Moreover, all three EOs exhibited potential for mucous membrane irritation, even at 0.5% [*v*/*v*] [82]. These findings underscore the significant toxicological risks associated with EOs, warranting thorough evaluation prior to any intended application [82]. Acute poisoning from EOs typically occurs due to accidental ingestion of undiluted oils, often leading to symptoms like rapid breathing, seizures, nausea, vomiting, and, in rare cases, death [83]. Dermatological reactions vary based on factors such as the type of compounds (like aldehydes and phenols), quality of the oil, method of application, dilution, and skin condition [83]. Due to EOs’ ability to easily penetrate the blood–brain barrier, there is a potential effect on the central nervous system after systemic absorption. Concerns during pregnancy include the risk of chemical compounds crossing the placental barrier, potentially affecting fetal development and increasing the risk of miscarriage [83]. Additionally, some EOs have been associated with endocrine-disrupting effects, particularly lavender and tea tree oils, which can activate estrogen receptors and potentially impact hormonal balance [83] (Figure 2).

The lack of regulations can result in EOs being diluted or adulterated to reduce production costs. This is a significant concern given the rapid growth of the commercial market for EOs, which was expected to increase in global market value from USD 17 billion to USD 27 billion by 2022 [84]. Approximately 80% of cases involving falsified EOs are detected on the market [84]. The adulteration of EOs often involves the addition of cheaper Eos or synthetic substances. Detecting this involves methods like comparing normalized areas of specific markers or analyzing the enantiomeric composition of chiral components [85]. Another common form of adulteration is diluting the EO with vegetable oils. This type of adulteration is more challenging to detect because it does not alter the qualitative composition or the normalized percentage of markers, making absolute quantitative analysis necessary [85]. The therapeutic effectiveness of EOs is undermined by the absence of quality standards and the ongoing issue of adulteration [84]. Adulteration of edible oils frequently leads to impurities that make them unsuitable for human consumption. There have been reported cases where adulterated oils caused severe health issues [86], e.g., edible oil adulterants such as argemone oil and butter yellow can increase the risk of gallbladder cancer [87]. Despite EOs being widely valued in various industries for their applications, their susceptibility to adulteration poses the risk of adverse health effects. Electronic nasal sensors present a promising solution for detecting such adulteration [88]. The development of MIPs for EO applications is a promising area of research, as it offers the potential for more efficient, selective, and sustainable extraction and purification methods [35,36]. By designing MIPs to target specific compounds in EOs, researchers can improve the overall quality and value of extracted materials, ultimately contributing to the advancement of various industries that rely on EOs, such as cosmetics, pharmaceuticals, and food production [89].

However, there are challenges related to MIP conformation such as the target molecule’s structure and conformation, its size, and flexibility, which can result in low affinities and heterogeneous binding sites within the MIP, inadequate diffusion of the target molecule through the MIP, and difficulties in effectively removing the template after the imprinting process [9]. The challenges associated with macromolecule imprinting can be solved by the advancement of surface-based and epitope imprinting techniques, as well as the utilization of nanoscale MIPs with surface-exposed imprinted sites. These nanosized MIPs offer a high surface-to-volume ratio, enhancing interactions with proteins and facilitating analyte diffusion to electrode surfaces. This feature is critical for achieving detectable signals in electrochemical platforms [90,91]. Additionally, MIPs face challenges with electropolymerization and mass production due to heterogeneous binding sites, and issues with bulk polymerization which hinder their compatibility with electrochemical detection methods [11]. The primary challenge in using MIPs for real biological samples, like blood, is the interference from abundant proteins such as serum albumin, which are present at much higher concentrations compared to the target proteins [11]. Saliva, blood, blood serum, urine, and other bodily fluids contain markers that are significant for biomedical applications [92,93]. Detecting low concentrations of biomarkers in complex matrices is technically challenging [93]. Catalytic and affinity-based biosensors are predominantly employed to detect certain biologically active substances within these biological samples [92]. While computational methods show potential in MIP development, no study has yet used them for the entire MIP preparation process [94]. Most studies employ these methods for specific steps, indicating the need for further development before they can be widely adopted as a standard pre-research procedure [94]. However, these methods would be beneficial for reducing expensive trial-and-error experiments and gaining a deeper understanding of the processes demonstrated at each stage [94].

### 3.4. Nanotechnological Approaches for the Preparation of Molecularly Imprinted Polymers

Nanotechnology plays a crucial role in developing nanoscale drug delivery tools, involving various methods to administer traditional drugs, recombinant proteins, vaccines, and nucleotides. Novel types of DDS are liposomes, proliposomes, microspheres, gels, prodrugs, cyclodextrins, nanoparticles, exosomes, and others [17].

In principle, molecular imprinting can be extended to larger molecules and even nanometer- and micrometer-sized objects like bacteria and viruses. However, it is important to note that nanomaterials, such as nanoparticles (NPs) or nanorods, differ from molecular materials as they consist of assemblies with varying molecular weights, shapes, and properties [95]. The NPs are frequently used as templates due to their ease of preparation and high symmetry. The removal of these nanomaterials is critical, with gold or silver NPs often preferred for their stability and ability to undergo electrochemical dissolution [95]. This dissolution allows for the quantification of re-uptaken NPs based on oxidation charge. For non-conductive nanomaterials, alternative detection methods such as capacitive measurement and mass change will need to be developed and tested [95]. Additionally, NPs serve as matrix support for a MIP shell, requiring synthesis tailored to the templates’ and functional monomers’ properties. Surface modifications of these NPs are often necessary to facilitate effective MIP polymerization and enhance the detectability of the MIP by improving its physical or chemical properties [96] (Table 1).

Also, careful evaluation of the surface chemistry of the NPs is essential to ensure that the MIP shell/layer forms very precisely, with high fidelity to the template, maximizing binding site specificity. Ultimately, these methods can significantly increase the sensitivity and selectivity of molecularly imprinted sensors, enabling their application in complex systems.

## 4. Conclusions

The significant potential of MIT in DDS using EOs plays a crucial role in innovative drug development. Different MIT application areas lead to the ability to enhance EO therapeutic efficacy and selectivity. The application of nanotechnology in MIT preparation leads to innovative technique development for optimizing DDS. By proposing models for investigating MIT-based EO delivery systems’ efficiency, this review highlighted the potential for impactful translational research in this field.

The MIPs present the potential for more efficient, selective, and sustainable extraction and purification methods of EOs compared to other drug delivery systems. However, MIPs face several challenges, including issues with target molecule structure and conformation, low affinities, heterogeneous binding sites, inadequate diffusion, difficulties in template removal, electropolymerization, mass production, and interference from abundant proteins in real biological samples. These challenges can be addressed by advancing surface-based and epitope imprinting techniques, utilizing nanoscale MIPs with surface-exposed imprinted sites, and further developing computational methods to streamline the MIP preparation process.

From clinical trials [74,75,76,77,78,79] investigating the therapeutic potential of essential oils (EOs), such as those evaluating lavender, bergamot, and chamomile, it became evident that leveraging MIPs in EO-based drug delivery systems (DDS) could offer significant advantages over traditional DDS like nanoparticles, liposomes, and nanogels. MIPs provide higher selectivity and specificity, as well as enhanced stability under various environmental conditions, making them particularly suitable for the sustained and controlled release of therapeutic agents. Furthermore, the reusability and cost-effectiveness of MIPs present a distinct advantage, reducing environmental impact and overall treatment costs.

The current state of MIT in EO-based drug delivery underscores the need for further research and development to realize the full potential of this innovative approach in applications.

## Figures and Tables

**Table 1:** Types of nanoparticles applied in molecular imprinting technology.

Structures Used for the Formation of Nanoparticles (NPs)	Description of Major Achievements
Polymeric	Widely used for the extraction and pre-concentration of both small and macromolecules from complex samples, e.g., tramadol and haloperidol [97,98].
Silica	A novel synthesis strategy using silica particles and controlled PEG addition which significantly reduced non-specific binding sites and improved MIP selectivity for lysozyme, increasing the imprinting factor from 2.1 to 9.1. This approach, which employed hydrophilic silica nanocores and click chemistry for AFCTP, was also applied to imprinted bovine hemoglobin, demonstrating its general applicability [99].
Carbon	Graphene oxide was used to synthesize a double-sided magnetic(M) MIP for the selective recognition of microcystins, incorporating Fe_3_O_4_ NPs coated with diphenylethene and acrylamide MIP, anchored to both sides of the GO sheets. This material enabled a magnetic solid-phase extraction procedure with an enrichment factor of 2000, limiting the quantification from 0.1 to 2.0 ng·L^−1^, and with recoveries of 84% to 98%, showing superior analytical performance and the potential for environmental microcystin removal [100].
Gold	The MIPs with AuNPs were developed for the selective detection of dimetridazole. The AuNPs were synthesized by reducing HAuCl_4_ and were coated with 3-propyl-1-vinylimidazolium bromide, an ionic liquid used as a monomer for MIP synthesis. These imprinted AuNPs were then applied to modified glassy carbon electrodes, resulting in a sensor with a detection limit of 5.0 × 10^−10^ mol·L^−1^ for dimetridazole, successfully tested on food samples [101].
Magnetic	A novel strategy for extracting and pre-concentrating bisphenol A from milk using a MIP with MNPs’ core synthesized through reversible addition-fragmentation chain transfer polymerization was reported. The MIP, containing β-cyclodextrin and 4-vinyl pyridine as functional monomers and bisphenol A as the template, achieved highly selective cavities, superior selectivity, a detection limit of 3.7 µg·L^−1^, and high recovery rates of 97% to 99% [102].

## Data Availability

No new data were created or analyzed in this study. Data sharing is not applicable to this article.
